# Factors affecting adherence to a high-risk surveillance protocol among patients with Li-Fraumeni syndrome

**DOI:** 10.1186/s13053-023-00259-z

**Published:** 2023-08-11

**Authors:** Kaylee A. Underkofler, Martha H. Thomas, Christina J. Taylor, Christa L. Mazur, Sarah H. Erickson, Kari L. Ring

**Affiliations:** 1 Emily Couric Clinical Cancer Center, University of Virginia, 1240 Lee St, 22903 Charlottesville, VA USA; 2 Department of Radiology and Medical Imaging, University of Virginia, Charlottesville, VA USA

**Keywords:** Li-Fraumeni syndrome, Cancer, Health behavior, Risk management, Genetics

## Abstract

**Background:**

High-risk surveillance for patients with Li-Fraumeni syndrome (LFS) has shown a stage shift and improved overall survival, but is demanding. Our objective was to evaluate surveillance adherence in a population of patients with LFS presenting for high-risk care.

**Methods:**

A retrospective analysis of surveillance adherence of adult patients with LFS at a single institution was performed. Adherence was defined by the duration from initial University of Virginia (UVA) LFS clinic visit to the time of first missed surveillance test. Two-sample t-tests and ANOVA tests were used to identify factors associated with duration of adherence.

**Results:**

A total of 42 patients were evaluated in the UVA LFS clinic between 2017 and 2021. Of these, 21 patients met inclusion criteria. At the time of review, 6 patients (29%) were up to date with high-risk surveillance recommendations. The mean duration of adherence was 17 months. Female sex was found to be associated with longer duration of adherence (mean 21 mo vs. 3.5 mo for males, p = 0.02). A personal history or active diagnosis of cancer was also associated with increased adherence (p = 0.02). However, neither age (p = 0.89), geography (p = 0.84), or known family history of LFS (p = 0.08) were associated with duration of adherence.

**Conclusion:**

Female sex as well as a personal history of cancer were associated with longer duration of adherence to recommended high-risk surveillance among patients with LFS. Identification of barriers to surveillance will be essential moving forward to increase adherence and promote early detection of cancer, thereby reducing the morbidity and mortality of LFS.

## Introduction

Li-Fraumeni syndrome (LFS) is a rare, yet highly penetrant hereditary cancer syndrome, that confers a 50% risk of cancer by age 31 and a near 100% lifetime risk of cancer [1, 2]. It is typically caused by a germline pathogenic variant (PV) in *TP53*, a well-known tumor suppressor gene, and has a reported prevalence ranging from 1 to 3,555 in patients with a family history of LFS associated cancers to 1 in 5,476 in population based genomic studies [3, 4]. A PV in *TP53* can cause cancer is nearly every organ of the body, though the classic LFS associated malignancies include premenopausal breast cancer, soft tissue and bone sarcomas, brain tumors, hematologic malignancies, and adrenocortical carcinomas [5].

A diagnosis of LFS is established via genetic testing, though patients meeting all three clinical criteria without a known pathogenic *TP53* variant are also considered to have the disorder. These criteria include (1) a sarcoma diagnosed under age 45 in the proband, (2) a first-degree relative with any cancer diagnosed under age 45, and (3) an additional first- or second-degree relative in the same lineage with any cancer diagnosed under age 45 or a sarcoma at any age [6].

Patients meeting the genetic or clinical definition of LFS are recommended to undergo a rigorous cancer surveillance protocol for the purpose of early cancer detection and thus reduced cancer-related morbidity and mortality. Surveillance protocols involve a multimodal approach and can include physical exams, imaging tests, and lab work. While several protocols have been proposed, evidence is greatest for the “Toronto protocol” [1, 7]. A modified version of the Toronto protocol for adults can be seen in Table 1. The founding investigators of this protocol followed a cohort of patients with LFS over an 11-year period and found that of those patients who did adhere to recommended surveillance, a greater proportion of tumors were found in premalignant stages compared to those who did not undergo surveillance, suggesting earlier detection. They also found that overall 5-year survival was 88.8% in those who underwent surveillance, compared to 59.6% in those who chose not to undergo surveillance.

While this evidence argues strongly in favor of surveillance due to early detection and survival benefits, the amount and frequency of tests required are demanding and can place a burden on patients that may limit adherence [8]. This recognition is important, because if a protocol is not feasible to a patient population, either it must be modified or barriers must be broken down to achieve its theoretical benefits. The Toronto protocol study reported a 90–100% patient adherence rate to recommended surveillance [7]. However, the authors noted that only 55% of patients initially chose to undergo surveillance, highlighting a highly selected patient population included in the trial. In another study based on patient surveys, 78% of patients reported they were adherent to surveillance recommendations, though recommendations were not uniform or defined [9]. Interestingly, another patient survey study out of Germany found that decision to undergo surveillance was impacted by satisfaction with genetic counseling, but not by sociodemographics, cancer history, or distress level [8]. There is otherwise limited data regarding adherence to surveillance among patients with LFS. The objective of this study was to assess adherence to a defined high-risk surveillance protocol for all patients with LFS presenting to a single institution to examine real world feasibility of such rigorous protocols outside of a clinical trial setting, as well as to predict which patients may be at greater risk of missing recommended surveillance.

## Methods

An IRB-waived (University of Virginia IRB for Health Sciences Research, Protocol 24,069) retrospective analysis of adult patients with LFS at the University of Virginia (UVA) was performed. In 2017, UVA established a high-risk clinic specifically for patients over 18 years of age with LFS. The charts of all patients presenting to this clinic between 2017 when the clinic opened and September 2021 were initially reviewed. September 2021 was chosen as an endpoint to allow all patients within the study at least 12 months of follow-up.

**Table 1:** High-risk cancer screening protocol recommended by the UVA LFS clinic, based on the modified Toronto Protocol.(1) wbMRI = whole body MRI, US= ultrasound, EGD= esophagogastroduodenoscopy, CBC= complete blood count, LDH= lactate dehydrogenase, 17-OHP= 17-hydroxyprogesterone, DHEAS= dehydroepiandrosterone

	UVA LFS Clinic Surveillance Protocol	Toronto Protocol
**Test**	**Frequency**	**Frequency**
wbMRI	annually	annually
Breast MRI (for females)	annually	annually, in addition to annual mammogram
Brain MRI	annually	annually
Abdominal US	annually	every 3–4 months
Pelvic US (for females with reproductive organs)	annually	every 3–4 months
Colonoscopy/EGD	every 2 years	every 2 years
Skin exam	annually	annually
Labs (CBC, LDH, ESR, 17-OHP, total testosterone, DHEAS, androstenedione)	not routinely assessed	every 3–4 months

Patients were excluded from this study for the following: not alive at time of chart review, possession of a genetic variant that was initially suspicious for LFS but had since been down-graded, clonal hematopoiesis of indeterminate potential (CHIP) status rather than possession of a true pathogenic variant, loss to follow-up, or unknown adherence due to out-of-system results. Many patients had tests performed outside of the UVA EMR system, but returned to clinic every 6 months and shared their results. Patients were considered lost to follow-up if they did not return to clinic, and it was unclear whether they were being followed elsewhere with good adherence or if they simply had poor adherence and were no longer being surveilled.

Surveillance adherence was defined as the duration from initial UVA LFS Clinic visit to the time of first missed test, measured in months. The tests recommended by the UVA LFS Clinic are based on the modified Toronto Protocol and include the following: annual whole body MRI (wbMRI), annual brain MRI, annual breast MRI in females, annual abdominal ultrasound, annual pelvic ultrasound if female reproductive organs are present, colonoscopy and esophagogastroduodenoscopy (EGD) every 2 years, and annual skin exam (Table 1) [5]. Patients were provided this list of recommendations upon their initial visit with the UVA LFS Clinic. Of note, females who have undergone mastectomy are no longer recommended to have an annual breast MRI, and those who have undergone hysterectomy and bilateral salpingoophorectomy are no longer recommended to have an annual pelvic ultrasound. Typically, when a wbMRI is done, the ultrasounds, brain MRI, and breast MRI are done 6 months later so imaging occurs at least every 6 months. A complete physical exam in LFS clinic is also performed every 6 months. Tests were ordered at the clinic appointment prior to when each test was due, and if performed within the UVA system, patients received phone calls from the system to schedule their tests. Patients were not responsible for remembering the recommended tests and calling to book those appointments independently unless they chose to have a test performed outside the UVA system. A missed test was defined as failure to obtain one of the recommended tests for any reason by the recommended date based on entry to the UVA LFS Clinic, plus or minus 2 months to allow for routine scheduling delays beyond the patients’ control. In addition to calculating time to first missed test, all types of missed tests for the duration of the follow-up period were counted and recorded for each patient, though only one of each test type was counted.

Variables of interest that were hypothesized to potentially affect adherence to this rigorous, high-risk cancer surveillance protocol and were available within a patient’s chart included age, sex, state of residence, personal history of cancer, family history of cancer defined as any known family member with a history of cancer (whether first-degree or beyond), family history of LFS, and medical insurance coverage.

Study data were collected and managed using REDCap electronic data capture tools hosted at UVA [10, 11]. REDCap (Research Electronic Data Capture) is a secure, web-based software platform designed to support data capture for research studies, providing (1) an intuitive interface for validated data capture; (2) audit trails for tracking data manipulation and export procedures; (3) automated export procedures for seamless data downloads to common statistical packages; and (4) procedures for data integration and interoperability with external sources.

IBM SPSS Statistics (version 28.0.1.1, Armonk, NY, USA) was used for statistical analysis. Two-sample t-tests and ANOVA tests, when appropriate, were used to assess associations between patient factors and duration of adherence.

## Results

A total of 42 patients were initially evaluated in the UVA LFS clinic and had a chart review performed. A down-graded variant led to exclusion of 1 patient, 2 were excluded for CHIP status, 6 were lost to follow-up or excluded due to inaccessibility of outside records and thus unknown adherence, and 12 were excluded due to entry to the clinic after September 2021. This led to a total study population of 21 patients.

Among the 21 patients included, the median age was 46. All were English-speaking. Women accounted for the majority of the study population (n = 17, 81%). **Most patients were Caucasian (n = 13, 62%), 10% were Black (n = 2), 14% were Asian (n = 3), and 14% declined to state a race or ethnicity in their medical record (n = 3).** Most patients resided within the state of Virginia (n = 16, 76%). All others presented from bordering states, with the exception of 1 who resided in Florida. Regarding personal cancer history, most patients had a prior diagnosis of cancer (n = 11, 52%) or an active diagnosis (n = 2, 10%). No history of cancer was observed in 8 patients (38%). Nearly every patient had a family history of cancer (n = 20, 95%), as well as at least one relative with a known LFS diagnosis (n = 15, 71%). All patients in the study possessed medical insurance (n = = 21, 100%). Demographic data can be visualized in Table 2.

**Table 2:** Demographics of the study population

Patient Characteristic	Subgroup	n (%)
Total		21 (100%)
Age (years) *median 46*	< 40 40–60 > 60	6 (29%) 12 (57%) 3 (14%)
Sex	Male Female	4 (19%) 17 (81%)
Race	Caucasian Black Asian Unknown	13 (62%) 2 (10%) 3 (14%) 3 (14%)
State	Virginia Other	16 (76%) 5 (24%)
Cancer History	Active cancer Prior diagnosis Never diagnosed	2 (10%) 11 (52%) 8 (38%)
Family History of Cancer	Yes No	20 (95%) 1 (5%)
Family History of LFS	Yes No	15 (71%) 6 (29%)
Medical Insurance Coverage	Yes No	21 (100%) 0 (0%)

All 21 patients were followed for at least 12 months. Beyond 12 months, patients were followed for varying lengths of time based on the date of their first UVA LFS Clinic appointment to the time of chart extraction: 14 patients were followed for at least 24 months, 11 were followed for at least 36 months, 10 were followed for at least 48 months, and 3 were followed for at least 60 months. The average length of follow-up was 39 months for all patients, 41 months for females, and 26 months for males.

At the time of review, 6 patients (29%) were up to date with high-risk surveillance recommendations. Of those who were up to date, the median age was 47. All were female (n = 6, 100%), 4 resided within Virginia (67%), 5 had a prior diagnosis of cancer (83%) and 1 had no personal history of cancer (17%), 5 had a family history of cancer (83%), and 2 had a known family history of LFS (33%). Demographic data for patients who were up to date with surveillance can be visualized in Table 3.

**Table 3:** Demographics of patients up to date with the recommended high-risk screening protocol

Patient Characteristic	Subgroup	n (%)
Total		6 (100%)
Age (years) *median 47*	< 40 40–60 > 60	2 (33%) 3 (50%) 1 (17%)
Sex	Male Female	0 (0%) 6 (100%)
Race	Caucasian Black Asian Unknown	6 (100%) 0 (0%) 0 (0%) 0 (0%)
State	Virginia Other	4 (67%) 2 (33%)
Cancer History	Active cancer Prior diagnosis Never diagnosed	0 (0%) 5 (83%) 1 (17%)
Family History of Cancer	Yes No	5 (83%) 1 (17%)
Family History of LFS	Yes No	2 (33%) 4 (67%)
Medical Insurance Coverage	Yes No	6 (100%) 0 (0%)

**Fig. 1:**
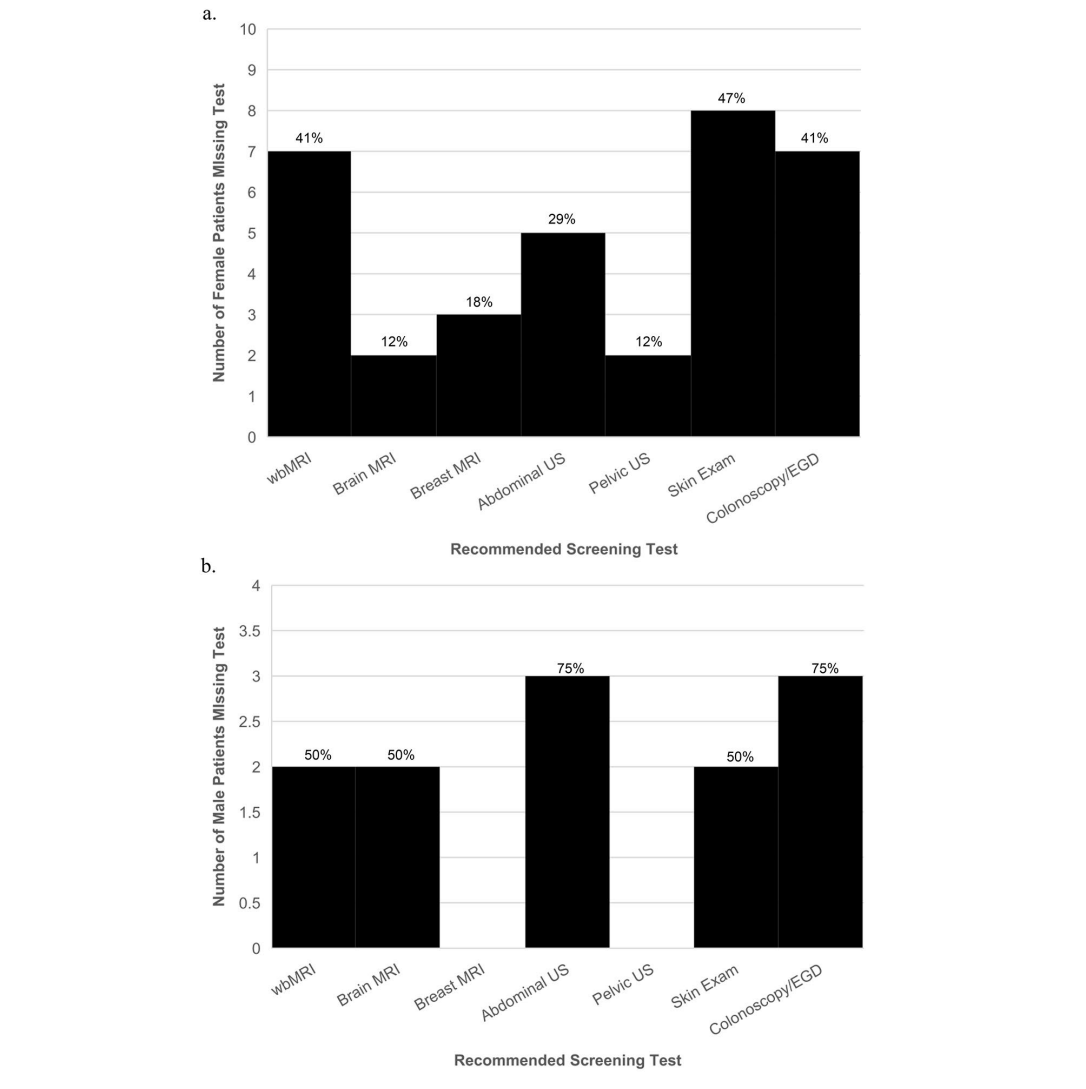
Most commonly missed tests of those recommended in a high-risk surveillance protocol for (**a**) females, and (**b**) males with LFS in the study population. The graph illustrates the number and percent of patients who missed each test at any point in their surveillance period. Breast MRI and pelvic ultrasound are not applicable for male patients. wbMRI = whole body MRI, US = ultrasound, EGD = esophagogastroduodenoscopy

Of the tests recommended for high-risk surveillance, those most commonly missed by the entire study population included colonoscopy & EGD (n=10, 48%), annual skin exam (n=10, 48%), and whole-body MRI (n=9, 43%) (Fig. 1). When stratified by sex due to differing surveillance recommendations, colonoscopy and EGD remained one of the most missed tests for both groups.

The mean duration of adherence to high-risk surveillance, defined as the time from initial presentation to LFS clinic to the first missed recommended test, was 17 months, with 11 of the 21 patients remaining adherent for the first 12 months (52%). Of the 14 patients who were followed for 24 months, 6 (43%) remained adherent throughout their surveillance period. Similarly, those followed for 36 months had a 36% adherence rate, those followed for 48 months had a 40% adherence rate, and those followed for 60+ months had a 33% adherence rate. Female sex was found to be associated with longer duration of adherence (mean 20 mo vs. 3.5 mo for males, p=0.02). Females were adherent for 49% of their length of follow-up and males were adherent for 13% of length of follow-up on average. A personal history or active diagnosis of cancer was also associated with increased adherence (mean prior history 28 mo vs. active diagnosis 16 mo vs. no history 2.5 mo, p=0.02). However, neither age (p=0.89), race (p=0.36), geography (p=0.84), or known family history of LFS (p=0.08) were associated with duration of adherence (Table 4).

**Table 4:** Mean duration of adherence based on multiple patient factors

Patient Characteristic	Subgroup	Mean Duration of Adherence (mo)	p-value
Total mean (range)		17 (0–64)	
Age (years)	< 40 40–60 > 60	16 15 29	0.89
Sex	Male Female	3.5 20	0.02
Race	Caucasian Black Asian Unknown	23 0 6 14	0.36
State	Virginia Other	18 15	0.84
Cancer History	Active cancer Prior diagnosis Never diagnosed	16 28 2.5	0.02
Family History of LFS	Yes No	10 34	0.08

Importantly, the COVID-19 pandemic fell in the middle of the follow-up period for this study. Of the 12 patients who were followed starting prior to the pandemic, only 2 missed tests and disrupted their adherence during 2020 after the shutdown. The remainder either became non-adherent prior to the pandemic (n = 5) or continued to follow their surveillance schedule through the pandemic and beyond (n = 5). Of the 6 patients who were up to date at the time of chart extraction, 5 started surveillance prior to the COVID-19 shutdown and only 1 started after.

## Discussion

The results of this study suggest that a minority of patients with LFS are able to adhere to the demanding high-risk cancer surveillance protocol recommended. While 52% achieved adherence for the first 12 months, there was a general trend of decreasing adherence with longer follow-up and only 29% were up to date at the time of chart review. Female sex was associated with longer duration of adherence, which may be surprising because women in the general population are typically less adherent to screening colonoscopy recommendations than men, though it is noteworthy that there were only 4 males in our study sample [12]. A personal history of cancer was also associated with longer duration of adherence. Notably, while the 2 patients with active cancer diagnoses did have a roughly average length of adherence (14 and 18 months) compared to others in the study, neither were up to date with recommended surveillance at the time of chart review, likely due to prioritizing their active cancer treatment. No other patient factors were identified that could predict who may follow recommendations for an extended period. Residing in the state of Virginia, for example, was not associated with longer duration of adherence compared to those that resided out of state. It seems reasonable to hypothesize that geography could contribute to greater adherence if being closer to UVA, where the LFS Clinic and the only hospital in the region able to perform wbMRI are located, made it easier for patients to complete their tests. However, this was not found to be the case, and in fact, one of few patients who were up to date lived farthest in the state of Florida. One could also hypothesize that insurance status could affect adherence. In our study population, 100% of the patients were insured, and thus insurance status itself could not be analyzed as a factor contributing to adherence. However, type of insurance could be examined in the future, as simply having insurance does not necessarily mean coverage is adequate to cover expenses for a specific patient, and some insurance companies may cover a greater percentage of cost.

In assessing the tests most commonly missed of those recommended, colonoscopy and EGD were at the top of the list for all patients. This pattern was similarly seen among both males and females (Fig. 1). Consistent with published literature in both the general population and in other high-risk populations such as those with Lynch Syndrome, colonoscopy and EGD are often subject to decreased adherence [13, 14]. These are invasive and uncomfortable procedures that often require preparation and anesthesia of some sort, and require a patient to be accompanied to their appointment. Skin exam was missed as frequently as colonoscopy and EGD among all patients. This is likely due to the fact that many patients receive dermatology follow-up outside of the UVA system and are responsible for scheduling appointments on their own, thus decreasing adherence. WbMRI was also commonly missed. Several patients in the study were significantly delayed in completing their wbMRI by as much as a year, with insurance issues cited as a reason in their charts. WbMRI is also very limited in geographic availability, with UVA University Hospital serving as one of the only sites in the region to offer it, likely contributing to decreased adherence as well.

The adherence rate of 29% within this study is far lower than those reported in prior studies. For example, in the Toronto protocol study, a 90–100% adherence rate was noted [7]. However, this was among the 55% of their total study population that chose to undergo surveillance and was in the context of a trial. Our results may be lower because they account for all patients with LFS in a real world clinical setting, not just those choosing to enroll in a clinical trial, which is likely a highly motivated patient population. Furthermore, it is unclear whether patients in the Toronto protocol study received more prompting or more frequent follow-up than patients may receive in the UVA LFS clinic. The patient survey study with a 78% adherence rate is limited by patient report of adherence, which may not be accurate, and is further limited by the fact that recommendations were not defined or uniform among all patients being surveyed, potentially explaining the difference between our results [9].

It is worth noting that the favorable outcomes seen as a result of surveillance using the Toronto protocol, including stage shifts and improved mortality rates, resulted when using the original, more rigorous protocol initially proposed than the modified Toronto protocol version that has since been proposed by the authors and is more in line with that being used in the UVA LFS clinic [1, 7]. Therefore, it is unclear without further research whether use of the modified protocol, and adherence to it, will result in the same benefits.

A strength of this study was access to data of an unselected population of patients with LFS, meaning that all adult patients known to the UVA system with LFS were eligible for initial review, thus reducing selection bias. Additionally, all data extracted from patient charts was recorded by the same physician and genetic counselor who run the LFS clinic, thus information was documented in a uniform manner with few missing elements.

This study is not without limitations. First, LFS is a very rare disease, thus our study sample size for evaluating adherence at a single institution was small. However, the Toronto protocol study is one of the largest studies available in a LFS population and included only 89 patients, thus for a rare disease we were able to achieve a reasonably-sized sample for study [3]. Second, by utilizing patient records retrospectively for data collection, we were limited in the variables we were able to study to those factors that would be available within the chart for every patient. It would have been interesting to investigate socioeconomic status or the extent of insurance coverage for surveillance tests and the effect each of these had on adherence, for example, as well as patient-identified barriers. However, these are not documented in a consistent manner within the medical record, and thus could not be evaluated in this study. It is likely that only insured patients with high health literacy present for high-risk care at this time based on the clinic population. Third, the average duration of adherence may be skewed by the fact that some patients presented to the UVA LFS Clinic earlier than others. For this reason, patients that presented within 12 months of the time of chart review were excluded, and adherence rates based on duration of follow-up were calculated and reported in the results section. Furthermore, the average duration of follow-up among males was shorter than for females (26 months versus 41 months, respectively), calling into question the validity of female sex being associated with longer duration of adherence. Despite differences in average length of follow-up, the observation may stand because males duration of adherence in relation to their duration of follow-up was disproportionately lower than that among females. Finally, the impact of COVID-19 on surveillance adherence should not be overlooked. The first major pandemic-related health systems shutdown occurring from March 2020 to roughly June 2020 fell in the middle of the follow-up window for this study. While it is favorable that the majority of patients that were adherent at the start of the pandemic were able to continue with their scheduled surveillance despite the shutdown, there was a difference in adherence noted between those who were followed prior to 2020 and who started after 2020. It is possible this discrepancy is related to patient avoidance of healthcare settings or other similar barriers as a consequence of COVID-19, though it is unclear. There is good evidence for patient avoidance of healthcare during the initial phases of the pandemic, but less is known about whether this tendency has continued to now, almost 3 years from the primary shutdown [15]. It would be interesting to see if this pattern was observed in other institutions with similar high-risk surveillance programs.

In conclusion, a minority of patients with LFS remain adherent to a rigorous, high-risk cancer surveillance protocol for extended periods of time within the UVA system. Longer duration of adherence seems to be associated with female sex and a personal history of cancer, while age, state of residence, and family history of LFS are not associated. Future directions include examining patients with LFS who undergo recommended risk reducing procedures, such as mastectomy, and factors that influence their decisions. Additionally, identification of barriers to surveillance will be essential moving forward. Our group is actively studying patient-reported outcomes in LFS to identify some of these barriers, specifically the role of screening fatigue, with the hope that this knowledge may lead to interventions designed to increase surveillance adherence. Only when we increase adherence to recommended surveillance will the goal of early detection of cancer, and thus a reduction in the morbidity and mortality of LFS, be realized.

## Data Availability

The datasets generated and/or analyzed during the current study are not publicly available due privacy and ethical concerns but are available from the corresponding author on reasonable request.

## List of abbreviations

LFS: Li-Fraumeni syndrome
PV: pathogenic variant
UVA: University of Virginia
CHIP: clonal hematopoiesis of indeterminate potential
wbMRI: whole body MRI
EGD: esophagogastroduodenoscopy
